# An A2 β-casein infant formula with high sn-2 palmitate and casein phosphopeptides supports adequate growth, improved stool consistency, and bone strength in healthy, term Chinese infants: a randomized, double-blind, controlled clinical trial

**DOI:** 10.3389/fnut.2024.1442584

**Published:** 2024-08-14

**Authors:** Xiao-Yang Sheng, Wiola Mi, Qing Bin Yuan, Bryan Yanwen Liu, Virgilio Carnielli, Yi Bing Ning, Alexandra W. C. Einerhand

**Affiliations:** ^1^Department of Developmental Behavioral Pediatric and Children Healthcare, Xinhua Hospital, School of Medicine, Shanghai Jiao Tong University, Shanghai, China; ^2^Bunge Nutrition, Shanghai, China; ^3^Junlebao Nutrition Research Institute, Shijiazhuang, China; ^4^College of Biotechnology, East China University of Science and Technology, Shanghai, China; ^5^Division of Neonatology, Polytechnic University of Marche and ‘G. Salesi’ Children’s Hospital, Ancona, Italy; ^6^Einerhand Science and Innovation, Alkmaar, Netherlands

**Keywords:** infant nutrition, sn-2 palmitate, casein phosphopeptides, A2 β-casein, adequate growth, gastrointestinal tolerance, stool consistency, bone strength

## Abstract

The aim of this randomized, double-blind, controlled trial was to examine the effects of infant formula on the growth, stool consistency, and bone strength of infants (*n* = 120) over a period of 4 months. The investigational group was fed an A2 β-casein cow’s milk infant formula containing casein phosphopeptides (CPP) and high sn-2 palmitate (54% of total palmitate at sn-2). The control group was fed a standard cow’s milk formula without CPP and with low sn-2 palmitate (29% of total palmitate at sn-2). The third group was fed human milk (HM) (*n* = 60). All three groups had similar baseline characteristics, and maintained similar BMI, sleep habits, and growth rates in body weight and length throughout the study. However, compared to the control group, infants in the investigational and human milk groups had significantly: (i) greater body length at 90, 120, and 150 days of age; (ii) greater growth rate in head circumference from 30 to 60 days of age, with larger head circumference at 60 days of age; (iii) larger daily stool frequency at 60, 90, and 120 days of age; (iv) softer stool at 60, 90, and 120 days of age; (v) higher bone quality index and bone speed of sound at 150 days of age; (vi) fewer hours of crying at 60 and 90 days of age; (vii) less abdominal distention, burp, and flatus at 60, 90, and 120 days of age; and (viii) less constipation at 90 days of age. At other time points, no significant differences were observed between the three groups. No serious adverse events (AEs) related to the study products were reported, and significantly fewer infants in the investigational and HM groups experienced at least one AE compared to the control group. The study suggests that the A2 β-casein formula with high sn-2 palmitate and CPP supports adequate growth, is well tolerated, and may have beneficial effects on stool consistency, gastrointestinal comfort, crying duration, and bone density, comparable to HM.

**Clinical trial registration:** https://clinicaltrials.gov/, NCT04749290.

## 1 Introduction

Human milk (HM) is widely acknowledged to be the optimal source of nutrition to support overall growth and development in infants. However, there are circumstances when breastfeeding may not be feasible or preferred, leading to the use of alternative milk-based infant formulas (IF) to meet the nutritional requirements of infants (1). It is therefore crucial to optimize the nutritional composition of these IFs to ensure that they adequately support infant growth and development.

Fat is a significant component of both HM and IF, serving as a vital energy source for infants. Fat accounts for approximately 50% of the energy content of HM, and occurs primarily in the form of triglycerides (~98–99% of HM lipids) (2–4). Palmitic acid is the primary saturated fatty acid in HM. It usually represents about 20–25% of HM fatty acids. Approximately 60–70% of palmitic acid present in HM is esterified to the sn-2 position of the glycerol backbone (sn-2 palmitate) (5–7). Most conventional infant formulas utilize vegetable oils as the primary source of fat, replacing bovine milk fat as the most common source since the mid-twentieth century. Unlike HM, most palmitic acid present in vegetable oils is esterified to the sn-1 (sn-1 palmitate) and sn-3 positions (sn-3 palmitate), with less than 20% esterified to the sn-2 position (5, 8).

The benefits of using sn-2 palmitate in IF has been well documented. More than 20 intervention studies have been conducted on infants to assess the effects of high sn-2 palmitate IF on infant growth, digestion, and other health outcomes (9–30). These studies have shown improved energy and gastrointestinal (GI) comfort, with reduced calcium soaps formation (2, 5, 31). According to a position paper written by the European Society for Pediatric Gastroenterology, Hepatology, and Nutrition (ESPGHAN) Committee on Nutrition, studies have generally shown that infant formulas with sn-2 palmitate can support adequate growth and development in infants (2). Infant formulas with a combination of high sn-2 palmitate, fructo- and galacto-oligosaccharides, and partially hydrolyzed whey proteins have been shown to manage digestive problems in infants, including colic, regurgitation, and constipation (11–13). A combination of high sn-2 palmitate and oligofructose has been shown to reduce stool palmitate soaps, calcium soaps, and total soaps, while promoting softer stools (9, 10). Calcium and fatty acid soaps have been shown to be positively related to stool hardness (32).

Calcium is a crucial mineral, especially during infancy, as it plays a pivotal role in the development and maintenance of strong bones (33). Adequate calcium intake during childhood helps promote optimal bone mineralization, ensuring that bones grow strong and reach their full potential in terms of density and strength. Previous trials of both full-term and preterm infants have demonstrated that high sn-2 palmitate IF enhances calcium absorption and bone mineralization compared to traditional IF, and reduces calcium soaps formation in infant stool (10, 14–18, 23, 24, 34).

Calcium absorption and bioavailability within bone tissues may also be improved by supplementation with casein phosphopeptides (CPP), which are derived from milk’s casein protein, and are rich in phosphorus. This effect is well supported by *in vitro* and animal studies, as well as some human investigations, although to our knowledge, none of these have been conducted on infants. CPP can bond with calcium ions within the low pH environment of the GI tract, preventing the precipitation of calcium phosphate in the intestine, and thereby facilitating the transport of less soluble forms of calcium. CPP’s effect on calcium absorption has potential for improved bone formation and prevention of bone resorption (35–42).

Cow’s milk proteins, including β-casein, are important ingredients to add to IF, because they provide certain essential amino acids for growth and development, and contribute to the overall nutritional profile of the formula (43). β-casein is an important protein found in cow’s milk. β-casein is expressed in cow’s milk as two primary genetic variants–A1 and A2–but is typically only expressed as the A2 variant in HM. IF based on A2 β-casein, excluding A1 β-casein, may therefore more closely mimic HM, and may help to maintain optimal infant growth and development (43).

After ingestion, A1 β-casein undergoes hydrolysis to form β-casomorphin-7 (BCM-7), which activates gastrointestinal opioid receptors, leading to a decrease in GI motility and an increase in GI transit time (44–46). This suggests that A1 β-casein may contribute to GI intolerance. On the other hand, A2 β-casein does not produce BCM-7, indicating that milk containing solely A2 β-casein might improve GI motility and reduce GI symptoms compared to milk containing both A1 and A2 β-caseins (44–46).

There is extensive evidence from animal trials (46, 47), and emerging evidence from seven human intervention trials linking the intake of milk containing A2 without A1 β-casein to GI improvements (48–54). The subjects of two of these seven human studies were children: toddlers (age 12 to 26 months) (48) and preschoolers (age 5 to 6 years) (53). However, thus far, no studies have been conducted on infants. Therefore, there is a need for clinical studies to further explore the benefits of A2 β-casein excluding A1 β-casein to optimize the nutritional composition of IFs and ensure that they can adequately support infant growth and development.

Based on the aforementioned scientific evidence supporting the potential benefits of A2 β-casein, high sn-2 palmitate, and CPP, our hypothesis is that a combination of these ingredients in a new IF may lead to improved infant health outcomes (e.g., growth, tolerance, and digestive comfort). We also hypothesize that the addition of CPP may augment the effects that sn-2 palmitate has already been shown to exert on calcium absorption. By incorporating these ingredients into the formula, we aim to replicate some of the beneficial aspects of HM. Hence, this study was designed to determine the growth, stool consistency, and bone strength of infants receiving an investigational formula with A2 β-casein, high sn-2 palmitate, and CPP for a duration of 4 months, and compare the results to those of infants receiving commercially available control formula with low sn-2 palmitate without CPP, as well as to those of infants exclusively receiving HM. To our knowledge, this is the first clinical study conducted on an A2 β-casein IF with high sn-2 palmitate and CPP, providing important information on its safety and effects on growth, gut, and bone health.

## 2 Materials and methods

### 2.1 Study participants and study design

This double-blind, randomized, controlled trial (Clinicaltrials.gov: NCT04749290) included 120 participants at a clinical site with two branches in Jin Hua, ZheJiang Province, China. Participants were born between 37 and 42 weeks of gestation, and were enrolled in the study at about 30 days of age. The calculation of the study group size (60 participants per group) is based on established parameters from previous publications on stool characteristics (10, 12, 14, 15, 18). In addition, 60 infants exclusively fed human milk were recruited for comparison. Parents or guardians provided written informed consent before enrollment. The study protocol and informed consent forms were drafted in accordance with the ethical principles that have their origin in the Declaration of Helsinki, and were consistent with Good Clinical Practice (GCP) and applicable regulatory requirements. The study was approved by the Shanghai Nutrition Academy Medical Ethical Committee.

**Figure 1:**
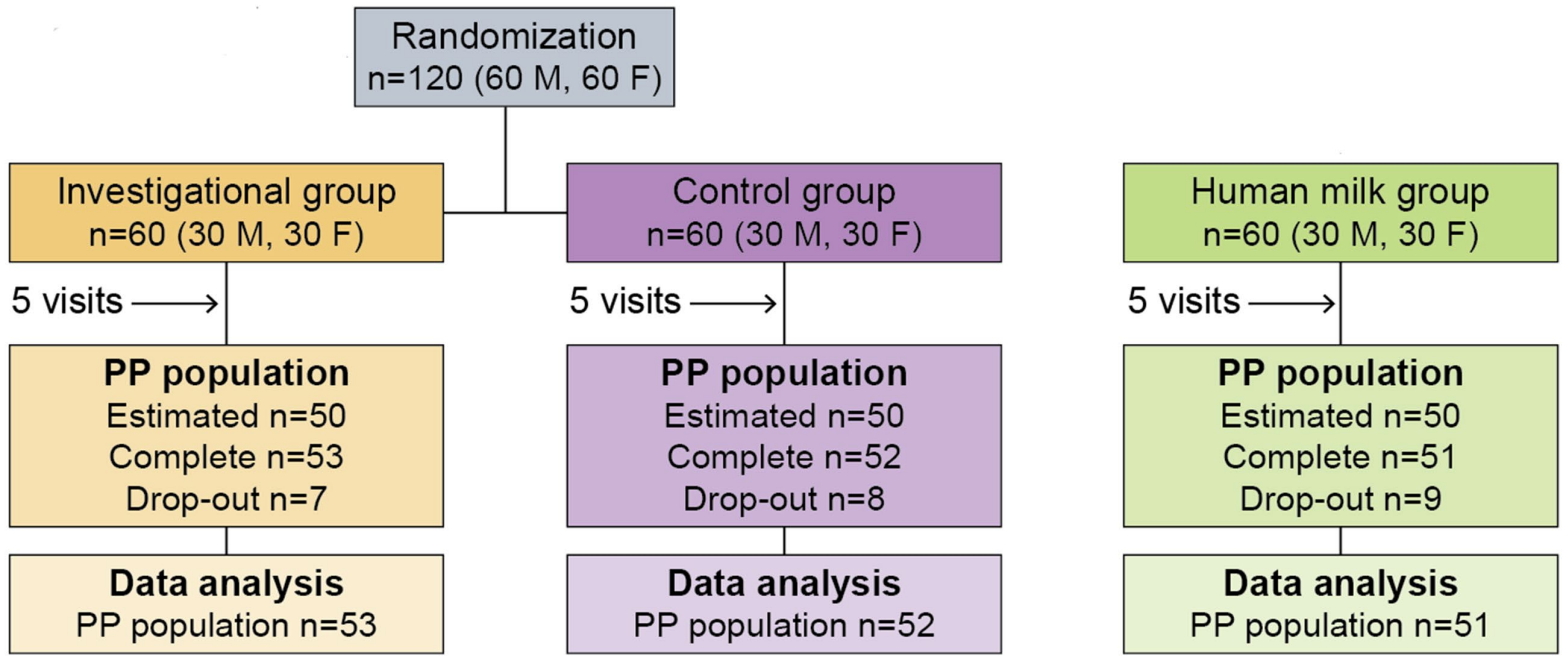
Flow chart of clinical study design.

The study design included a pre-screening and screening process, until 180 participants were selected to participate in the study. Inclusion and exclusion criteria are described in Supplementary Table S1. The study design (Figure 1) required three study groups:

- Group 1 (investigational group), which would receive A2 β-casein cow’s milk infant formula (ZhiZhen, Junlebao dairy, Shijiazhuang, China) with high sn-2 palmitate (54% of total palmitate at sn-2) (Betapol® Plus, Bunge Loders Croklaan) and CPP throughout the duration of the study.
- Group 2 (control group), which would receive commercially available A1 & A2 β-casein cow’s milk infant formula with low sn-2 palmitate (29% of total palmitate at sn-2) (Junlebao dairy, Shijiazhuang, China) without CPP throughout the duration of the study.
- Group 3 (human milk group), which would exclusively receive HM throughout the duration of the study.

The study design (Figure 1) required that participants make a total of five visits over the course of 4 months, at 30 ± 3 (enrollment), 60 ± 3, 90 ± 3, 120 ± 3, and 150 ± 5 days of age.

Of the 180 total participants, 120 were either exclusively formula-fed, or partially formula-fed with a minimum daily intake of 600 mL of formula in addition to HM, and had been formula-fed for a minimum of 3 days before randomization. Each formula-fed infant was randomly assigned to either Group 1 or Group 2 through the generation of a randomization schedule. Stratified randomization was used to control for sex, so that each of the two groups would end up consisting of an even split of 30 male and 30 female infants (*n* = 60). The remaining 60 of the 180 total participants were exclusively fed HM for at least 7 days prior to enrollment. These infants were designated as Group 3, which was also consisted of an even split of 30 male and 30 female infants.

### 2.2 Study measurements

At the first study visit (enrollment) participants’ demographic information, anthropometric data at birth, and medical history were collected from medical records. Additional information on parental demographics, medical history, and substance use were gathered during an interview using a standardized form. During each of the five study visits, parents reported infants’ gastrointestinal complaints (if any) from the previous 24-h period. The severity of GI complaints (abdominal distention, burp, flatus, diarrhea, constipation, colic, diaper dermatitis, hogback) was scored on a 0–5 point scale (0 = none, 1 = very light, 2 = light, 3 = moderate, 4 = severe, and 5 = very severe). Parents also reported the number of instances (if any) of infant vomiting or nausea from the previous 24-h period. Parents photographed each stool passed in the 24-h period prior to each of the five visits, and investigators evaluated stool consistency (A = watery, B = soft, C = formed, D = hard), stool amount (1 = smear; 2 = up to 25%; 3 = 25–50%; 4= > 50% of feces in the reference area), and stool color (I = yellow; II = orange; III = green; IV = brown; V = meconium; VI = clay-colored) according to the Infant Stool Form Scale (55). Mean stool frequency for each study group was calculated for the previous 24-h period prior to each of the five visits by dividing the total number of bowel movements by the total number of infants remaining in the study group at each time point. Duration of crying (24-h recall) and seven-day recall on sleep patterns were also collected at each visit via questionnaires.

Anthropometric measurements were collected at enrollment and each subsequent visit by trained study personnel following standardized procedures. Infants (naked) were weighted twice on calibrated electronic scales. Infant length was measured in triplicate using a standard measuring board (Shanghai Betterren). To measure head circumference, a non-stretchable measuring tape was used in triplicate.

After enrollment, parents were asked to record subjects’ frequency and severity of adverse events, medication use, and visits to a health care professional, which was discussed during each study visit. Parents also reported intake of HM, investigational formula, and complementary food (if any). All of the collected information was used by investigators to evaluate the subjects’ tolerance to the study products.

Bone strength was evaluated via determination of Bone Quality Index (BQI) at enrollment (30 ± 3 days) and at the final study visit (150 days±5). Bone Quality Index (BQI) was measured using two ultrasound (Ultrasound Bone Densitometer BMD-A3) parameters: speed of sound (SOS) and broadband ultrasound attenuation (BUA) (56). The formula for calculating BQI is:


$$BQI=\alpha^{*}\;SOS+\beta^{*}\;BUA\text{,}$$

where:

- α and β are temperature correction factors;
- SOS is the speed of sound, which represents the speed at which ultrasonic waves travel through bone tissue (57); and
- BUA is the broadband ultrasound attenuation, which measures the amount of ultrasound energy absorbed and scattered as it passes through bone (58).

The BQI calculation combines the values obtained from SOS and BUA to provide an overall assessment of bone quality and strength, and provides a holistic evaluation. Bone strength was also evaluated based on SOS on its own, as SOS gives an indication of bone density and microarchitecture. SOS has also been found to be positively correlated with gestational age, birth weight, length, head circumference, and tibial length of newborn infants (59).

### 2.3 Study products

The investigational formula (ZhiZhen, Junlebao Dairy) and control formula (commercially available IF, Junlebao Dairy) were manufactured per good manufacturing practices (ISO22000) and compliant with Chinese GB 10765–2010 (Table 1). The investigational and control formulas each had a similar caloric density, lipid level (26.2 g/100 g vs. 26 g/100 g), and DHA/ARA levels and ratio. The main differences between the two IFs were: (1) sn-2 palmitate level (investigational = 54% vs. control = 29%); (2) with CPP (investigational) vs. without CPP (control); and (3) A2 β-casein cow’s milk base (investigational) vs. regular A1 and A2 β-casein cow’s milk base (control). The calcium to CPP ratio in the investigational formula was 3:1, similar to previous studies using such ingredients to enhance calcium absorption in animals and humans (60, 61). The investigational and control formula were indistinguishable by taste or appearance. Both formulas were provided in powder form and packaged in coded tins. Parents or caregivers were asked to follow general feeding advice regarding appropriate feeding volumes per day, as printed on the formula label or as provided by their health care professional.

**Table 1:** Key compositions of investigational (Group 1) and control (Group 2) formulas.

Per 100 mL	Investigational IF^1^	Control IF^2^
Energy, kcal	64	65
Fat (total), g	3.4	3.4
Saturates, g	1.2	1.4
Monounsaturates, g	1.4	1.2
Polyunsaturates, g	0.8	0.8
Linoleic acid, mg	542	516
α-linoleic acid, mg	54	43
Arachidonic acid, mg	6.8	6.7
Docosahexaenoic acid, mg	5.4	5.0
Palmitic acid, mg	818	650
Sn-2 palmitate^4^, %	54	29
Protein, g	1.4	1.5
Whey protein, g	0.8	0.9
Casein, g	0.5	0.6
Carbohydrates, g	6.9	7.1
CPP^3^, mg	13.8	–
Calcium^3^, mg	41	49
Iron, mg	0.4	0.7
Zinc, mg	0.4	0.5

^1^Investigational IF: ZhiZhen, Junlebao Diary (information based on manufacturer nutritional label). Fat sources include Betapol®Plus (approximately 80% of total fat) and milk fat (approximately 20% of total fat). ^2^Control IF: commercially available infant formula, Junlebao Diary (information based on manufacturer nutritional label). Fat sources include vegetable oil (approximately 80% of total fat) and milk fat (approximately 20% of total fat). ^3^Ratio of calcium and casein phosphopeptide (CPP) = 3:1. ^4^Sn-2 palmitate percentage (%) has been analyzed according to Chinese GB standards (GB30604-2015). Sn-2 palmitate % represents sn-2 palmitic acid relative to total palmitic acids from all positions (sn-1, sn-2 and sn-3).

### 2.4 Study objectives and endpoints

The objectives of this study were to compare (over a period of 4 months) the growth, stool consistency, and bone strength of infants receiving:

- A2 β-casein, high sn-2 palmitate formula with CPP (Group 1);
- A1 and A2 β-casein, low sn-2 palmitate formula without CPP (Group 2); and
- HM (Group 3).

The calculation of the study group size is based on established parameters from previous publications on stool characteristics (10, 12, 14, 15, 18). A goal of 150 participants was set to complete the study. To cover a potential moderate drop-out rate of 15% in a clinical trial involving healthy term infants in China, the enrollment goal was set at 180 participants, providing a power of 80% in a two-tailed analysis of variance (ANOVA) with a significance level of 0.05.

The secondary objectives of this study were to compare the formula intake and tolerance (24-h dietary recall), crying duration (24-h recall), sleep patterns (seven-day recall), and medically diagnosed adverse events collected throughout the study period between the three groups.

### 2.5 Statistical analysis

Analysis of variance (ANOVA) was used for statistical testing for continuous outcomes unless specified otherwise. For stool amount, consistency, and ordinal tolerance data, study group comparisons were made using the Cochran–Mantel–Haenszel row-mean score test (62). Each time point was analyzed separately. Group differences for crying duration were evaluated using Cochran–Mantel–Haenszel row-mean score test (62). Group differences for bone quality index (BQI) and speed of sound (SOS) were evaluated using ANOVA.

Adverse events (AE) were reported by parents to the investigators, and coded for medical diagnosis, severity, and likely causality/relatedness to the study products. AE were independently verified by a medical monitor before unblinding of the study. Fisher’s exact test was used to compare the proportion of participants in each study group with AEs by body system and by event. In addition, the proportion of participants in each study group with at least one AE were compared.

A two-tailed significance level of 0.05 was used for statistical testing. Bonferroni adjustment was applied for multiple comparisons across the investigational (Group 1), control (Group 2), and HM (Group 3) groups.

All analyses were performed using SAS 9.4 software.

## 3 Results

### 3.1 Baseline characteristics

#### 3.1.1 Subject characteristics

There were no significant differences among study groups with respect to subjects’ age at enrollment, sex, birth history, and primary caregiver. Feeding habits before enrollment were well balanced between the two formula groups (Supplementary Table S2). The recruitment process and study flow of participants is summarized in Figure 1.

#### 3.1.2 Family and household characteristics

None of the parents or siblings of the 180 subjects had a history of asthma, allergic rhinitis/atopic keratoconjunctivitis, allergic dermatitis/atopic eczema, or allergic conjunctivitis (data not shown). Among each study group, 18.3–21.6% of subjects had smokers in their home (data not shown). However, there was no significant difference among study groups with respect to smoking exposure. In addition, there were no significant differences with respect to parental demographic information or medical history among the study groups (Supplementary Table S3). No significant group differences were observed with respect to ethnic background or socioeconomic status.

### 3.2 Feeding amount

The two formula-fed groups had been fed similar amounts at baseline, and maintained similar feeding habits throughout the study. The HM group did not consume any infant formula at baseline or throughout the study.

### 3.3 Anthropometric measurements

#### 3.3.1 Achieved body weight, length, head circumference, and BMI

Anthropometric measurements included body weight, body length, BMI, and head circumference. No significant differences were observed at enrollment (30 ± 3 days of age) with respect to any of these measurements across the entire study population (*n* = 180), nor were there any significant differences in these measurements between male and female participants.

**Figure 2:**
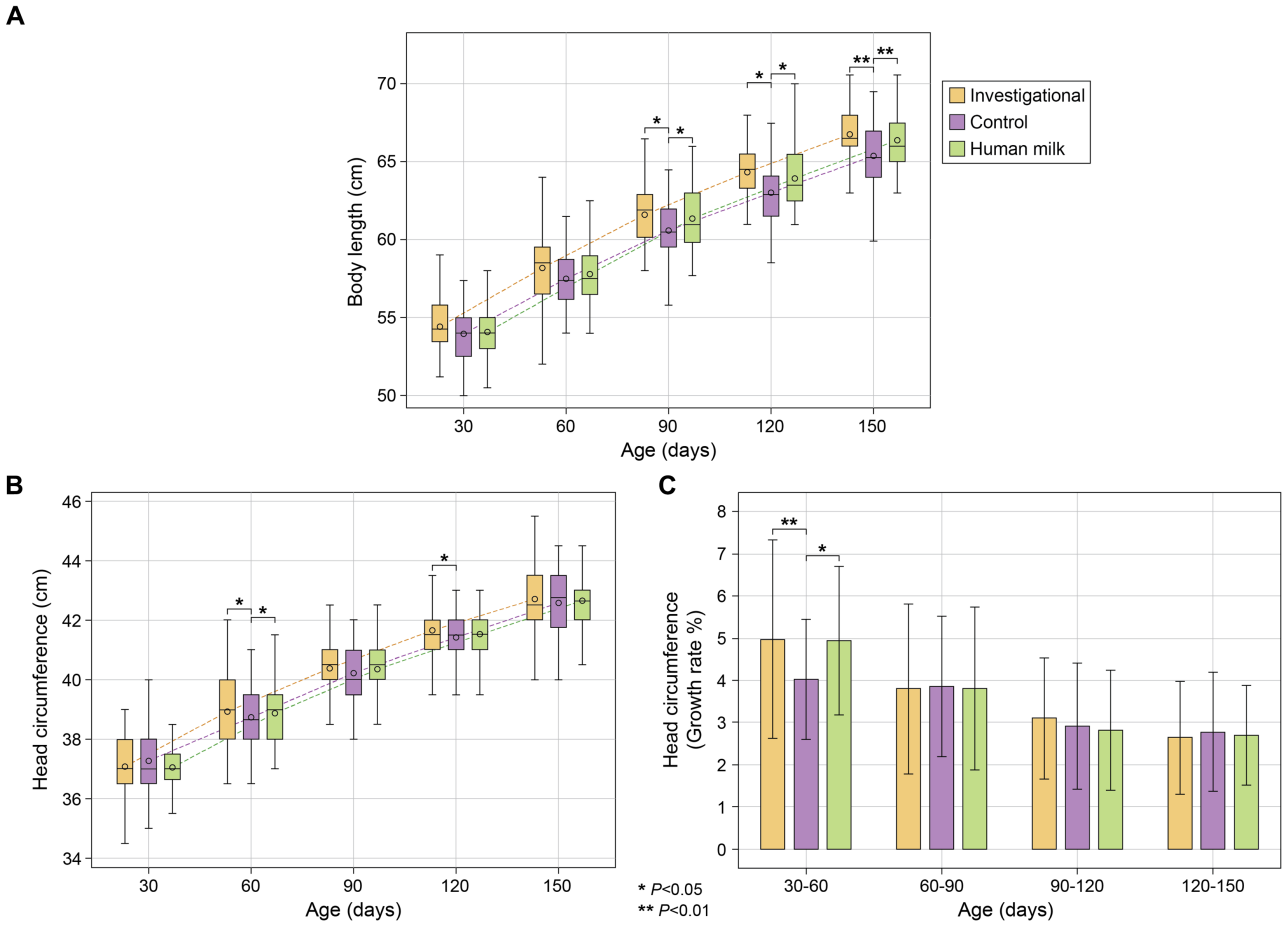
(**A**) Body length (cm); (**B**) head circumference (cm); and (**C**) average growth rate (%) of head circumference between five study visits. Body length (cm) and head circumference (cm) represent as mean ± standard deviation. Head circumference growth rate (%) as mean ± standard deviation.

Infants in the investigational group (Group 1) and the HM group (Group 3) had significantly longer body lengths compared to the control group (Group 2) at 90 (Group 1 *p* = 0.016 and Group 3 *p* = 0.013), 120 (Group 1 *p* = 0.012 and Group 3 *p* = 0.011), and 150 (Group 1 *p* = 0.008 and Group 3 *p* = 0.007) days of age (Figure 2A; Supplementary Table S4). Both the investigational group (Group 1) and the HM group (Group 3) showed significantly larger head circumference than the control group (Group 2) at 60 days of age (Group 1 p = 0.011 and Group 3 p = 0.016), and the investigational group had significantly larger head circumference compared to the control group at 120 days of age (*p* = 0.039; Figure 2B; Supplementary Table S4). The investigational group was comparable to the HM group in all anthropometric measurements at all study visits. No significant differences in body weight or BMI were observed across any of the three groups throughout the study (Supplementary Table S4).

#### 3.3.2 Growth rates of body weight, length, and head circumference between visits

All three groups (investigational, control, and HM) showed similar rates of growth in terms of body weight and length, with no significant differences observed between any of the three groups. With regard to head circumference however, participants in both the investigational (Group 1) and HM (Group 3) groups had faster growth rates from 30 to 60 days of age (Group 1 *p* = 0.008 and Group 3 *p* = 0.010; Figure 2C; Supplementary Table S5). After that, all three study groups maintained similar growth rates for the rest of the study.

### 3.4 Stool frequency and characteristics

**Figure 3:**
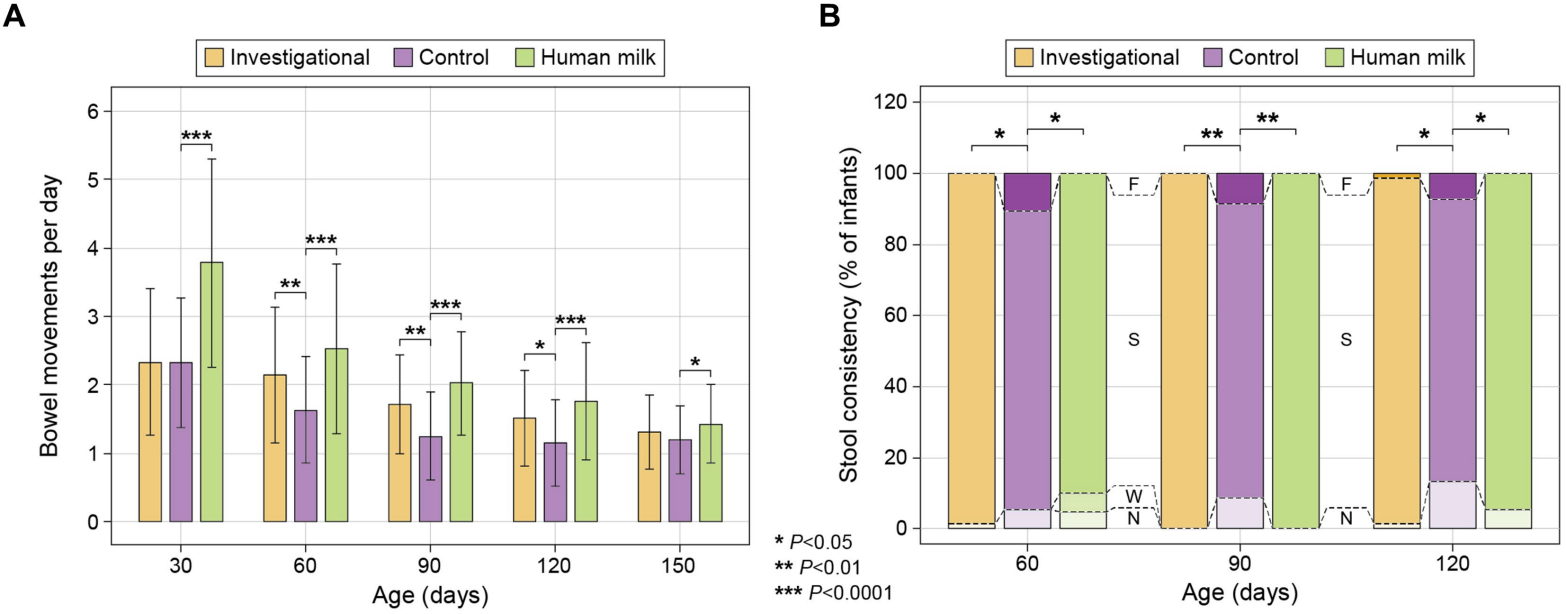
(**A**) Average stool frequency (mean ± standard deviation): calculated at each time point as the total number of bowel movements in the previous 24 h divided by the total number of infants remaining in the study group; and (**B**) Stool consistency of infants categorized as: No Bowel Movement (N), watery (W), soft (S), formed (F), or hard (H) (55). The percentage (%) of infants exhibiting each type of stool consistency is presented at 60, 90, and 120 days.

A 24-h recall questionnaire was completed by parents at each study visit to report infants’ stool frequency, and photographs were provided by parents for investigators to evaluate stool characteristics. No significant differences were observed at enrollment (30 ± 3 days of age) with respect to stool frequency or stool characteristics across the formula-fed infants (Groups 1 and 2). However, the exclusively human milk-fed infants (Group 3) had significantly greater daily stool frequency at the time of enrollment (30 ± 3 days of age) compared to the two formula groups (both *p* < 0.0001; Figure 3A; Supplementary Table S6). Participants in the HM group also had softer stool and lighter stool color compared to the two formula groups at the time of enrollment.

At 60, 90, and 120 days of age, participants in both the investigational group (Group 1) and the HM group (Group 3) had significantly greater daily stool frequency than the control group (Group 2) [(Group 1 at 60 days *p* = 0.008; 90 days *p* = 0.001; and 120 days *p* = 0.015) (Group 3 at 60, 90, and 120 days *p* < 0.0001)]. The HM group had significantly greater stool frequency than the control group at 150 days of age (*p* = 0.025; Figure 3A; Supplementary Table S6). At 60, 90, and 120 days of age, participants in both the investigational group and the HM group also had significantly softer stool than the control group [(Group 1 at 60 days *p* = 0.021; 90 days *p* = 0.004; 120 days *p* = 0.026) (Group 3 at 60 days *p* = 0.027; 90 days *p* = 0.003; 120 days *p* = 0.048)] (Figure 3B; Supplementary Table S6). Stool consistency of the three groups was similar at 150 days of age without significant differences (Supplementary Table S6). The HM group had greater stool quantity compared to the control group at 90 and 120 days, and lighter colored stool at 30 and 90 days (Supplementary Table S6). Notably, fewer infants in the investigational and HM groups had no bowel movements in the 24 h prior to the study visits at 90 and 120 days compared to the control group (Figure 3B; Supplementary Table S6).

### 3.5 Gastrointestinal symptoms

**Figure 4:**
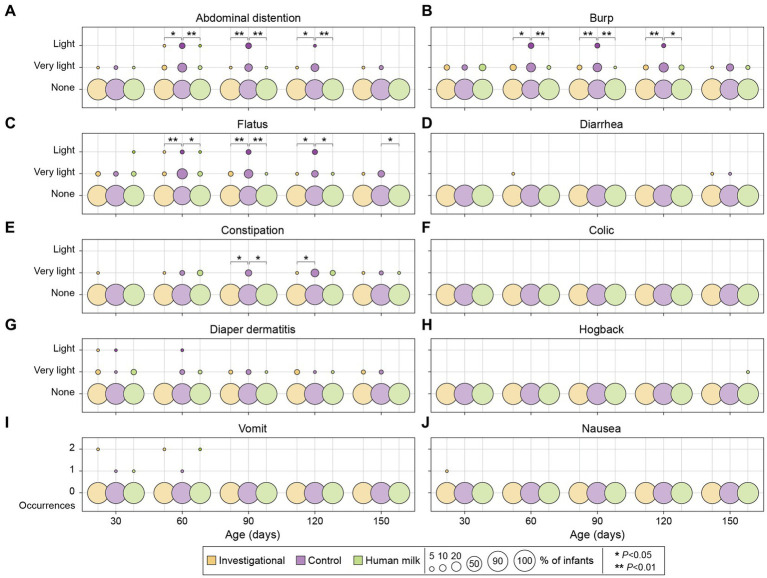
Gastrointestinal symptoms among investigational, control, and HM groups across five study visits. A 0–5 points scale was used to evaluate the degree of severity for abdominal distention, burp, flatus, diarrhea, constipation, colic, diaper dermatitis, and hogback **(A–H)**: 0 = none; 1 = very light; 2 = light; 3 = mild; 4 = moderate; 5 = severe. Vomiting and nausea were reported as number of occurrences **(I, J)**. Each circle represents the percentage (%) of infants who experienced the corresponding gastrointestinal symptoms.

A 24-h recall questionnaire was completed by parents at each study visit to report any gastrointestinal symptoms (e.g., vomiting or nausea) experienced by their infants. No symptoms ranked above a severity degree of 2 according to the 0–5 point scale (0 = none; 1 = very light; 2 = light; 3 = mild; 4 = moderate; 5 = severe) were reported at any point throughout the duration of the study. None of the infants experienced more than two occurrences of nausea or vomiting in the 24-h period leading up to any time point throughout the duration of the study. No significant differences were observed at enrollment (30 ± 3 days of age) with respect to GI symptoms across the entire study population (*n* = 180). The investigational and HM groups continued to have similar degrees of GI symptoms at all subsequent visits throughout the study. Compared to the control group (Group 2), participants in both the investigational (Group 1) and HM (Group 3) groups had significantly lower degrees of abdominal distention, burp, and flatus at 60, 90, and 120 days of age (Figure 4; Table 2).

**Table 2:** Significance values for comparison of gastrointestinal symptoms.

Outcome	Age (days)	*P*- value Investigational vs. Control	*P*- value HM vs. Control
Abdominal distention	60	0.014	0.006
	90	0.003	0.002
	120	0.015	0.006
Burp	60	0.010	0.001
	90	0.008	0.001
	120	0.009	0.012
Flatus	60	0.006	0.010
	90	0.008	0.001
	120	0.014	0.016

^1^Group differences were evaluated using Cochran–Mantel–Haenszel row-mean score test. Significance level for multiple comparisons was 0.05/3 = 0.017 with Bonferroni adjustment.

No significant differences in these symptoms were observed across the three groups at 150 days of age, with the exception of the HM group experiencing less flatus compared to the control group (*p* = 0.024; Figure 4). At 90 and 120 days of age, the investigational group experienced less constipation compared to the control group (90 days *p* = 0.022; and 120 days *p* = 0.026; Figure 4). At 90 days of age, the HM group also experienced less constipation compared to the control group (*p* = 0.019; Figure 4). There were no significant differences in the degree of diarrhea, colic, diaper dermatitis, hogback, vomiting, or nausea across the three groups during the study period (Figure 4; Supplementary Table S7).

### 3.6 Crying duration

**Figure 5:**
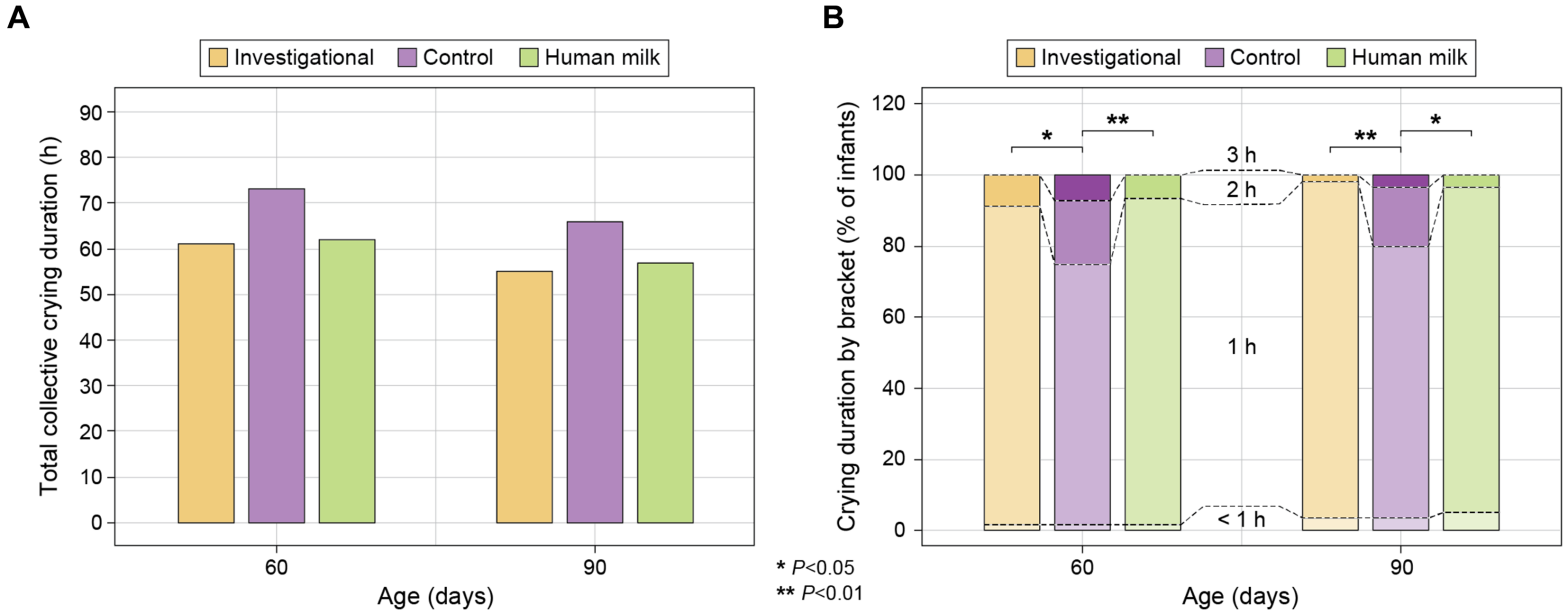
(**A**) Total collective crying duration in hours for each study group at 60 and 90 days (reported on 24-h recall surveys as estimates of <1, 1, 2, or 3 h), calculated as: [0*(Count for “<1 h” bracket)] + [1*(Count for “1 h” bracket)] + [2*(Count for “2 h” bracket)] + [3*(Count for “3 h” bracket)]; and (**B**) percentage (%) of infants falling within each estimated bracket of crying duration (<1, 1, 2, and 3 h) within each study group at 60 and 90 days.

A 24-h recall questionnaire was completed by parents at each study visit to report the crying duration in hours of their infants. Parents reported crying duration on the questionnaires by selecting from a list of estimated brackets: <1 h, 1 h, 2 h, or 3 h. Infants in the investigational (Group 1) and HM (Group 3) groups spent similar amounts of crying duration throughout the study, and both groups spent significantly fewer hours crying compared to the control group (Group 2) at 60 days (Group 1 *p* = 0.015 and Group 3 *p* = 0.009) and 90 days (Group 1 *p* = 0.007 and Group 3 *p* = 0.011; Figure 5). There was no significant difference in the number of crying hours across the three groups at 120 days and 150 days (Supplementary Table S8).

### 3.7 Sleep habits

A seven-day recall questionnaire was completed by parents at each study visit to report the sleep habits of their infants. All study groups had similar sleeping habits throughout the study. No significant differences across study groups were observed at enrollment or throughout the study (data not shown).

### 3.8 Bone density

**Figure 6:**
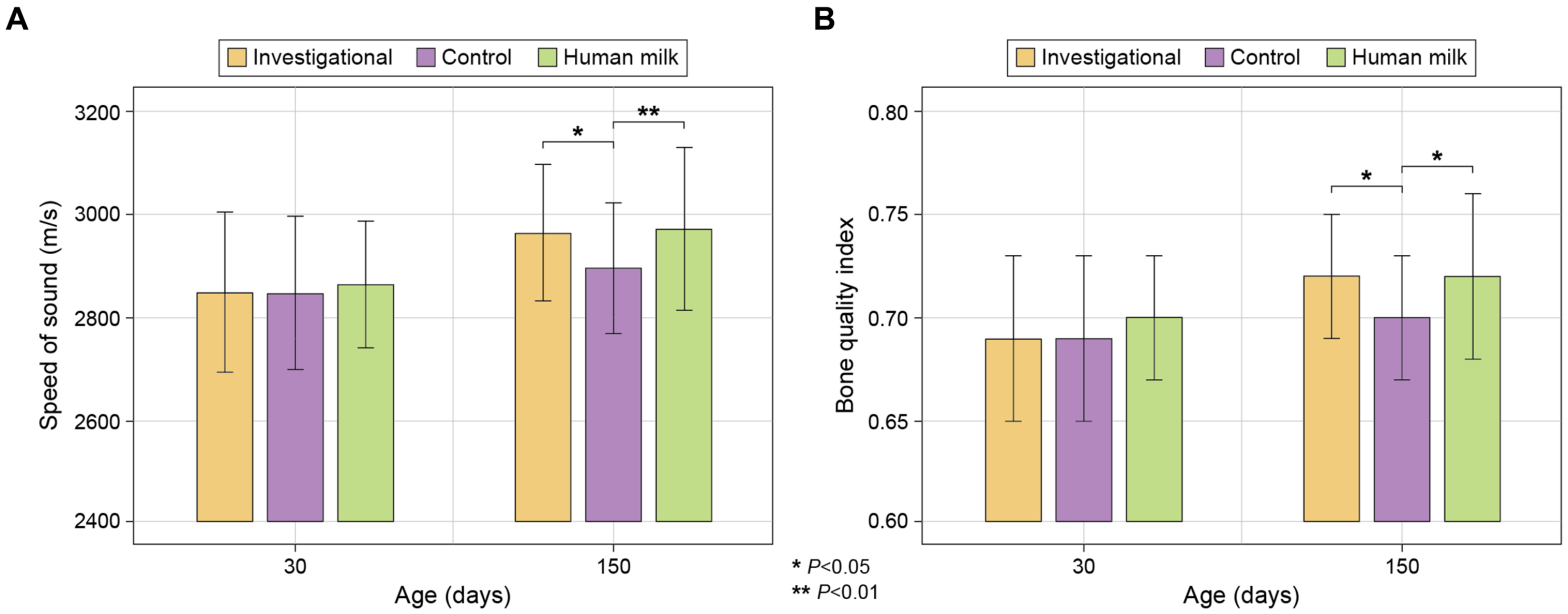
(**A**) Bone density by SOS (m/s); and (**B**) Bone Quality Index (BQI), calculated as BQI = α*SOS + β*BUA (2.2. Study Measurements). Data presented are mean ± standard deviation.

No significant differences were observed across the three study groups with respect to speed of sound (SOS) or Bone Quality Index (BQI) at enrollment (30 ± 3 days of age). At the last study visit (150 days of age), no significant differences in bone density were observed between the investigational (Group 1) and HM (Group 3) groups, but both of these groups had significantly higher SOS (Group 1 *p* = 0.013 and Group 3 *p* = 0.007) and BQI (Group 1 *p* = 0.011 and Group 3 *p* = 0.010) values than the control group (Group 2; Figure 6; Supplementary Table S9).

### 3.9 Adverse events

There were a total of 198 adverse events (AEs) reported during the study period (Supplementary Table S10). Investigators determined that none of these AEs were related to the study products. None of the AEs reported during the study were deemed serious. No subjects withdrew from the study as a result of any AE. Compared to the control group (Group 2), significantly fewer infants in both the investigational (Group 1) and HM (Group 3) groups experienced one or more AEs (Group 1 *p* = 0.028, and Group 3 *p* = 0.045) in general.

## 4 Discussion

### 4.1 Growth and gastrointestinal tolerance

The current study evaluates the effects of an A2 β-casein formula with high sn-2 palmitate and CPP on growth and tolerance in infants. Both the investigational and HM groups exhibited similar growth patterns concerning body weight, length, BMI, and head circumference. This indicates that the investigational formula adequately supported infant growth during the intervention period. The lower prevalence of GI symptoms–such as abdominal distention, burping, flatus, and constipation–in the investigational and HM groups compared to the control group suggests that the investigational formula may be better tolerated than the control formula. Lastly, the lower incidence of adverse events in the investigational and HM groups compared to the control group indicates that the tolerability of the investigational formula is closer to that of HM.

The results of this study are consistent with previous studies with regard to the safety and tolerability of high sn-2 palmitate in infant formulas (2, 5, 31). Although previous studies did not find differences among formula-fed groups with or without high sn-2 palmitate regarding GI tolerance (9, 10, 23, 31, 34), the current study demonstrated better gastrointestinal tolerance in the investigational and HM groups compared to the control group. This difference might be associated with A2 β-casein milk and its potential gastrointestinal benefits (48–54). Milk containing A2 β-casein (without A1 β-casein) has been reported to provide better gastrointestinal tolerance and comfort compared to regular A1 and A2 β-casein cow’s milk when consumed by toddlers and preschoolers (48, 53).

### 4.2 Digestive health

Digestive problems, such as a reduced capacity to digest fats, proteins, and lactose, often occur during the first few months of life as a result of the immature gastrointestinal tracts of newborns. These digestive problems are more likely to occur in formula-fed infants than those fed HM exclusively (63). The investigational group in our study received A2 β-casein IF without A1 β-casein and with high sn-2 palmitate in order to more closely imitate HM, thereby improving digestion, reducing GI symptoms, and improving stool characteristics. Indeed, compared to the control group, the stool frequency and stool consistency of the investigational group was significantly more comparable to those in the HM group. The lower prevalence of GI symptoms in the investigational group compared to the control group also suggests that the investigational formula may promote better digestive health.

Previous studies have demonstrated that high sn-2 palmitate IF can improve stool softness and frequency in infants compared to standard formula (9, 11–14, 18, 19, 29, 34). Furthermore, high sn-2 palmitate has been shown to reduce stool palmitate soaps, calcium soaps, and total soaps, while promoting softer stools (9, 10, 14, 16). As our investigational formula contained a much higher (54%) concentration of sn-2 palmitate than our control formula (29%), we hypothesize that the observed effects on stool characteristics in this study could be attributed at least in part to a reduction in calcium and/or fatty acids soap formation. This hypothesis is currently under further investigation through analyzing fecal samples from this clinical study.

Consumption of milk containing A2 without A1 β-casein has been linked to improvements in GI health in animal (46, 47) and human intervention trials in toddlers, preschool children, and adults (48–54). As elucidated in the introduction, β-casomorphin-7 (BCM-7)–the product of A1 β-casein hydrolysis–has been shown in human studies to activate GI opioid receptors, leading to decreased GI motility, increased GI transit time, and looser stool consistency, while animal studies suggest it may initiate inflammatory responses in the gut (44–47). All of these effects might be associated with digestive discomfort.

The current observations on stool softness, improved frequency, and reduced GI symptoms might be associated with synergetic effects of sn-2 palmitate and A2 β-casein without A1 β-casein. However, this hypothesis cannot be confirmed directly within the scope of the current study design. Future research can shed light on the mechanism of this potential synergy.

### 4.3 Crying duration

Here we show that the investigational group and the HM group had similar crying patterns throughout the study. Both groups experienced significantly fewer hours of crying compared to the control group at 60 and 90 days. The reduction in crying duration is an important finding, as excessive crying in infants can be distressing for both the infant and caregivers. If A2 β-casein-containing formulas can contribute to reduced crying and promote overall comfort in infants, it may be considered a beneficial option in infant nutrition. A2 β-casein has not been studied for its potential effects on crying duration in infants, whereas high sn-2 palmitate formula has previously been shown to improve crying and sleep patterns during the first weeks of life (19–22). However, we did not observe an improvement in sleep patterns as has been previously reported (21).

The potential underlying mechanisms that might influence GI comfort and, consequently, crying duration is discussed in Section 4.2. Further research may shed light on the complex mechanism of sn-2 palmitate and A2 β-casein without A1 β-casein on gut health and their influence on the system of neuroendocrine mediators and regulators.

### 4.4 Bone strength

Our evidence supports the notion that the investigational formula could play a significant role in promoting optimal bone development in infants, approaching the benefits associated with HM. Based on our speed of sound (SOS) and Bone Quality Index (BQI) evaluations, both the investigational and HM groups demonstrated superior bone quality compared to the control group after the intervention period, indicating that the investigational formula may positively influence bone development. Previous studies have shown that high sn-2 palmitate enhances calcium absorption and bone mineralization in both full-term and preterm infants (10, 14–16, 18, 19, 24, 34).

When triglycerides are consumed, the fatty acids bonded to the sn-1 and sn-3 positions are freed by digestive enzymes in the small intestine, leaving the fatty acid in the sn-2 position as a monoglyceride to be absorbed. Palmitic acid is more favorably absorbed when esterified in the sn-2 position as a monoglyceride than as a fatty acid freed from the sn-1 or sn-3 positions (6, 7). Free palmitic acid tends to complex with minerals such as calcium, and form insoluble soaps which cannot be absorbed in the small intestine, and are thus excreted in the feces. Therefore, high sn-2 palmitate results in higher palmitic acid and calcium absorption, leading to improved bone development (2, 5, 31).

Several adult and preclinical studies have demonstrated the effects of CPP on calcium absorption (35–42, 61), so the addition of CPP may also have contributed to the improvement in bone mineralization. However, to the best of our knowledge, no previous study has examined the effects of CPP in infants, so further research is needed to explore the precise role of CPP in supporting infant bone health. Although the observed effect on bone strength in this study cannot be directly attributed to CPP, we hypothesize that CPP, in synergy with sn-2 palmitate, can increase calcium absorption and thus achieve robust effects on infants’ bone development. This potential synergistic effect of CPP and sn-2 palmitate and the mechanism behind it deserve further investigation.

### 4.5 Limitations of the study

The strengths of this study include the well-balanced subject characteristics, rigorous data collection, and the inclusion of a HM group for comparison. However, limitations–such as the relatively short intervention period and the lack of long-term follow-up–should be considered when interpreting the results. Furthermore, when conducting the clinical study, limitations arose from the current practices in China, where it was not feasible to obtain a fully comparable control formula with identical ingredients. The study protocol required that the control formula be registered and already in the market for ethical committee approval. Due to these constraints, the study team had to work with an available commercial formula while ensuring compliance with ethical guidelines and regulatory requirements. While the control formula used in the study may not have been an exact match, every effort was made to find the closest commercially available analog to ensure the study’s scientific rigor and integrity.

## 5 Conclusion

The result of this randomized, double-blind, controlled trials support that the investigational formula–an A2 β-casein infant formula with high sn-2 palmitate and CPP–is well tolerated, supports adequate growth, significantly improves stool characteristics, and promotes bone health in Chinese full-term infants over a period of 4 months, compared to a control formula – standard cow’s milk formula without CPP and with low sn-2 palmitate.

The noteworthy similarities observed between the investigational and HM groups suggest that the investigational formula represents a promising step forward in emulating the benefits of HM. These findings contribute valuable evidence regarding the formula’s efficacy, and underscore the need for further research to explore its long-term effects and optimize its potential in infant nutrition.

## Data Availability

The datasets presented in this study can be found in online repositories. The names of the repository/repositories and accession number(s) can be found in the article/Supplementary material.
